# Development and Validation of HPTLC Method for the Estimation of Clotrimazole in Bulk drug and Tablet Formulation

**DOI:** 10.4103/0250-474X.57299

**Published:** 2009

**Authors:** Parul Parmar, Ankita Mehta

**Affiliations:** L. M. College of Pharmacy, Navrangpura, Gujarat-380 009, India

**Keywords:** HPTLC method of estimation, Clotrimazole, Tablets

## Abstract

A simple, precise, accurate and rapid high performance thin layer chromatographic method has been developed and validated for the determination of clotrimazole in bulk drug and tablet dosage form. The stationary phase used was precoated silica gel 60F_254_. The mobile phase used was a mixture of cyclohexane:toluene:methanol:triethyleamine (8:2:0.5:0.2 v/v/v/v). The detection of spot was carried out at 262 nm. The method was validated in terms of linearity, accuracy, precision and specificity. The calibration curve was found to be linear between 200 to 1000 ng/spot for clotrimazole. The limit of detection and the limit of quantification for clotrimazole were found to be 50 ng/spot and 200 ng/spot, respectively. The proposed method can be successfully used to determine the drug content of bulk drug and marketed formulation of tablet.

Clotrimazole is a broad spectrum antimycotic agent. Clotrimazole is chemically 1H-imidazole-1-[(2-chlorophenyl)diphenylmethyl][1]. Various analytical methods have been reported for the estimation of clotrimazole that include HPLC[2–8], UV/Vis spectrophotometric[9–13], colorimetric[14], differential pulse polarographic[15]. A few HPTLC methods[16–19] were also reported for the estimation of clotrimazole in creams and ointment formulation. However no method was reported for estimation of clotrimazole in tablet dosage form. The present study describes a simple, sensitive and precise HPTLC method for the estimation of clotrimazole in bulk drug and tablet dosage form.

Clotrimazole working standard was obtained as a gift sample from Relish Pharmaceuticals Limited, Ahmedabad, India. Silica gel 60F_254_ TLC plates (20×20 cm, layer thickness 0.2 mm E. Merck, Germany) were used as the stationary phase. All chemicals and reagents used were of analytical grade. Cyclohexane:toluene:methanol:triethyleamine (8:2:0.5:0.2 v/v/v/v) was used as mobile phase. Methanol was used as solvent. Tablets containing clotrimazole (equivalent to 100 mg clotrimazole) were purchased from a local pharmacy (Candid, Glenmark Pharmaceutical Ltd and Canesten, Bayer Pharmaceutical Ltd). A Camag HPTLC system comprising of Camag linomat IV semiautomatic sample applicator, Hamilton syringe (100 μl), Camag TLC scanner-3, Camag CATS4 software, Camag Twin trough chamber (10×10 cm) and ultrasonicator were used during study.

Working standard of clotrimazole (10 mg) was weighed accurately and diluted with methanol to obtain the final concentration of 40 μg/ml. The contents of twenty tablets were grounded to a fine powder. Weight equivalent to 100 mg of clotrimazole was transferred to conical flask and dissolved in 10 ml methanol. The solution was sonicated for 15 min and filtered using Whatman filter paper No 41 and residue was washed with methanol. The extracts and washings were pooled and transferred to a 25 ml volumetric flask and volume was made with methanol. Required dilutions were made to get 40 μg/ml of clotrimazole. TLC plates were prewashed with methanol. Activation of plates was done in an oven at 50° for 5 min. The chromatographic conditions maintained were precoated silica gel 60F_254_ aluminum sheets (10×10 cm) as stationary phase, cyclohexane:toluene:methanol:triethyleamine (8:2:0.5:0.2 v/v/v/v) as mobile phase, chamber and plate saturation time of 30 min, migration distance allowed was 50 mm, wavelength scanning was done at 262 nm keeping the slit dimension at 2×0.2 mm. A mercury lamp provided the source of radiation.

To prepare calibration curve, from standard solution of 40 μg/ml clotrimazole aliquots of 5, 10, 15, 20 and 25 μl were applied on the TLC plate. The TLC plate was dried, developed and analyzed photometrically as described earlier. The developed method was validated in terms of linearity, accuracy, specificity, limit of detection and limit of quantification, intra-day and inter-day precision and repeatability of measurement as well as repeatability of sample application (Table 1). For analysis of the formulation, 10 μl of the filtered solution of the formulation was spotted on to the same plate followed by development scanning. The analysis was repeated in two marketed tablet formulations. The content of the drug was calculated from the peak areas recorded.

**TABLE 1:** METHOD VALIDATION PARAMETERS

Parameters	Results
Linearity range (ng/spot)	200-1000
Correlation coefficient	0.9998
Regression equation (y=mx+c)	
Slope (m)	1.5007
Intercept (c)	370.08
Limit of detection (LOD)	50 ng/spot
Limit of quantitation (LOQ)	200 ng/spot
Precision (%RSD)	
Repeatability of application (n=5)	1.79
Repeatability of measurement (n=5)	0.69

A solvent system that would give dense and compact spot with appropriate R_f_ values was desired for quantification of clotrimazole in pharmaceutical formulations. The mobile phase consisting of cyclohexane: toluene:methanol:triethyleamine (8:2:0.5:0.2 v/v/v/v) gave R_f_ values of 0.22 (±0.05) for clotrimazole in bulk drug (fig. 1), and for clotrimazole in tablet formulation (fig. 2). The linear regression data (n=5) showed a good relationship over a concentration range of 200-1000 ng/spot for clotrimazole.

**Fig. 1:**
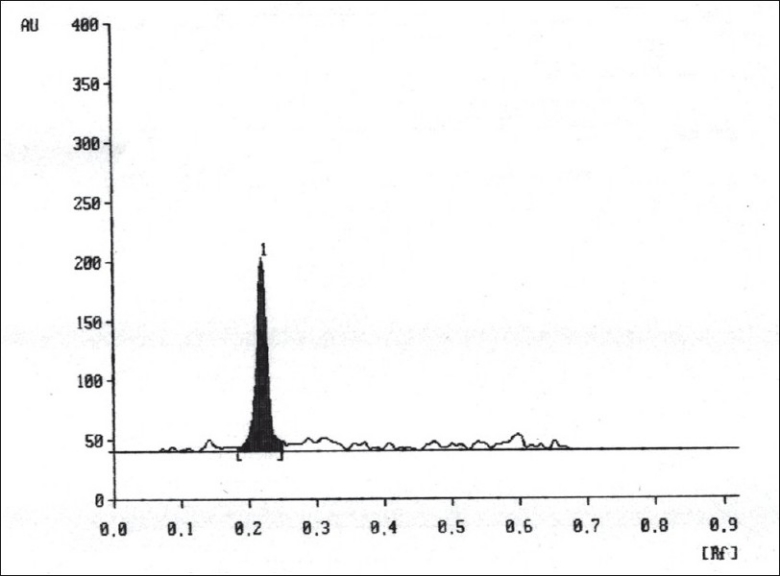
A typical chromatogram of clotrimazole

**Fig. 2:**
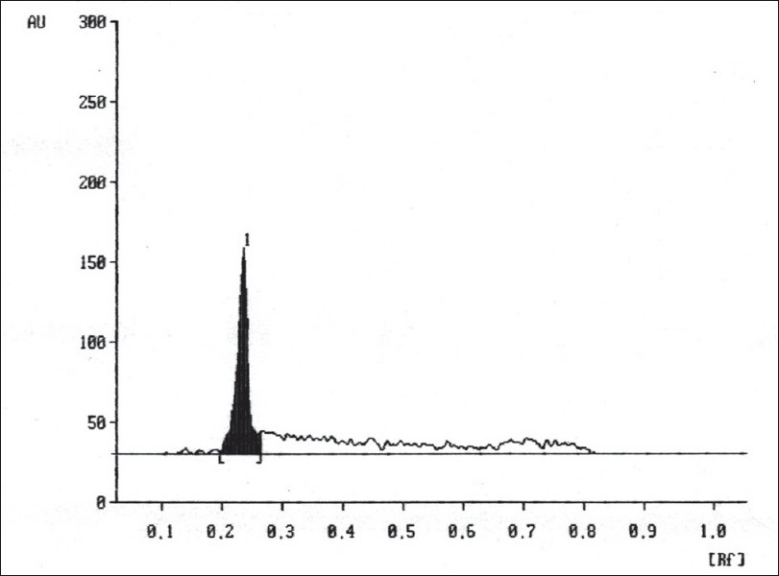
Chromatogram of clotrimazole in tablet dosage form

The limit of detection is the lowest amount of analyte in a sample that can be detected but not necessarily quantitated under the stated experimental conditions. Clotrimazole standard solutions (5 μg/ml) in the quantities of 5, 10, 15, 20, 25 and 30 μl were spotted on TLC plate, developed, dried in hot air and photometrically analyzed as described. The limit of detection was found 50 ng/spot (fig. 3). The limit of quantification (LOQ) is the lowest concentration of analyte in a sample that can be determined with the acceptable precision and accuracy under stated experimental conditions. Lowest concentration of calibration curve for standard clotrimazole was considered as LOQ. The LOQ was found 200 ng/spot (fig. 4).

**Fig. 3:**
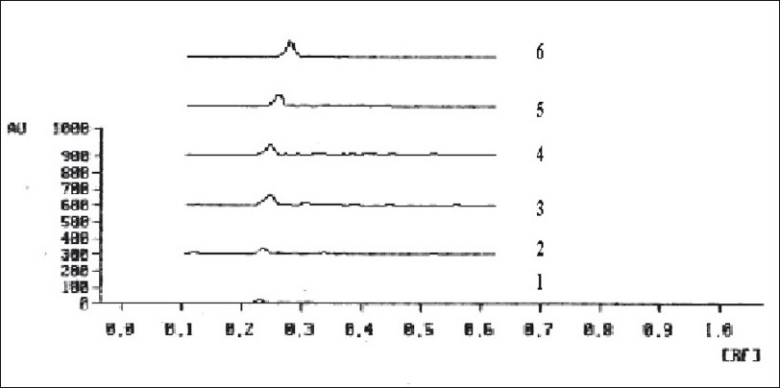
Limit of detection of clotrimazole Peaks 1 to 6 represent concentration range from 25 ng/spot to 150 ng/spot

**Fig. 4:**
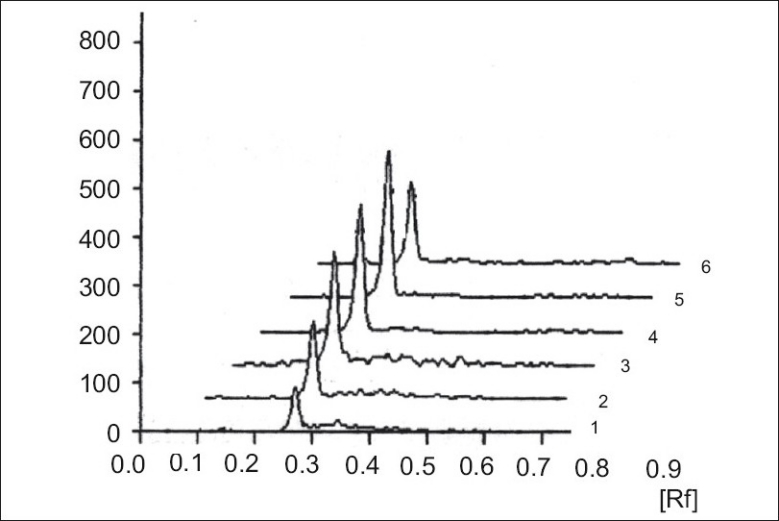
Chromatogram showing standard and clotrimazole from tablet formulation Peaks 1-5, 200-1000 ng/spot of standard clotrimazole, peak 6, 400 ng/ spot from tablet formulation

The intra-day precision was determined by analyzing standard solutions in the concentration range of 200 to 1000 ng/spot of clotrimazole for 3 times on the same day, while inter-day precision was determined by analyzing corresponding standards daily for 3 days over a period of one-week. The intra-day and inter-day coefficients of variation are in range of 0.61 to 3.36 and 1.17 to 4.44, respectively.

Repeatability of sample application was assessed by spotting 15 μl of drug solution 7 times on a TLC plate followed by development of plate and recording a peak area for 7 spots. The % RSD for peak area values of clotrimazole was found to be 1.79. Repeatability of measurement of peak area was determined by spotting 15 μl of clotrimazole solution on a TLC plate and developing the plate. The separated spot was scanned five times without changing the position of the plate and the % RSD for measurement of peak area of clotrimazole was 0.69. To confirm the specificity of the proposed method, the solution of the formulation was spotted on the TLC plate, developed and scanned. It was observed that the excipients present in the formulation did not interfere with the peaks of clotrimazole.

Recovery studies were carried out to assess accuracy parameters. These studies were carried out at three levels. Sample stock solution from tablet formulation of 40 μg/ml was prepared. Dilutions were made and recovery studies were performed. % Recovery was found to be within the limits as listed in Table 2. The low % RSD value indicated the suitability of the method for routine analysis of clotrimazole in pharmaceutical tablet dosage forms. The developed HPTLC technique is simple, precise, specific and accurate and the statistical analysis proved that the method is reproducible and selective for the analysis of clotrimazole in bulk drug and tablet formulations.

**TABLE 2:** RECOVERY STUDIES OF CLOTRIMAZOLE

Excess drug added to the formulation (%)	Theoretical content (ng)	Recovery (%)	% RSD
00	400	99.21	0.35
50	600	100.86	0.14
100	800	99.30	0.12
150	1000	100.21	0.076
